# Bergamot (*Citrus bergamia *Risso) fruit extracts and identified components alter expression of interleukin 8 gene in cystic fibrosis bronchial epithelial cell lines

**DOI:** 10.1186/1471-2091-12-15

**Published:** 2011-04-15

**Authors:** Monica Borgatti, Irene Mancini, Nicoletta Bianchi, Alessandra Guerrini, Ilaria Lampronti, Damiano Rossi, Gianni Sacchetti, Roberto Gambari

**Affiliations:** 1 Department of Biochemistry and Molecular Biology, University of Ferrara, Via Fossato di Mortara 74, Ferrara, 44121, Italy; 2 Department of Biology and Evolution, Agro-technological and pharmaceutical Resources (Agri-Unife), University of Ferrara, Corso Ercole I d'Este, Ferrara, 44121, Italy

## Abstract

**Background:**

Cystic fibrosis (CF) airway pathology is a fatal, autosomal, recessive genetic disease characterized by extensive lung inflammation. After induction by TNF-α, elevated concentrations of several pro-inflammatory cytokines (i.e. IL-6, IL-1β) and chemokines (i.e. IL-8) are released from airway epithelial cells. In order to reduce the excessive inflammatory response in the airways of CF patients, new therapies have been developed and in this respect, medicinal plant extracts have been studied. In this article we have investigated the possible use of bergamot extracts (*Citrus bergamia *Risso) and their identified components to alter the expression of IL-8 associated with the cystic fibrosis airway pathology.

**Methods:**

The extracts were chemically characterized by ^1^H-NMR (nuclear magnetic resonance), GC-FID (gas chromatography-flame ionization detector), GC-MS (gas chromatography-mass spectrometry) and HPLC (high pressure liquid chromatography). Both bergamot extracts and main detected chemical constituents were assayed for their biological activity measuring (a) cytokines and chemokines in culture supernatants released from cystic fibrosis IB3-1 cells treated with TNF-α by Bio-Plex cytokine assay; (b) accumulation of IL-8 mRNA by real-time PCR.

**Results:**

The extracts obtained from bergamot (*Citrus bergamia *Risso) epicarps contain components displaying an inhibitory activity on IL-8. Particularly, the most active molecules were bergapten and citropten. These effects have been confirmed by analyzing mRNA levels and protein release in the CF cellular models IB3-1 and CuFi-1 induced with TNF-α or exposed to heat-inactivated *Pseudomonas aeruginosa*.

**Conclusions:**

These obtained results clearly indicate that bergapten and citropten are strong inhibitors of IL-8 expression and could be proposed for further studies to verify possible anti-inflammatory properties to reduce lung inflammation in CF patients.

## Background

Cystic fibrosis (CF) pulmonary disease is an autosomal recessive disease caused by defective function of the CFTR protein product, a cAMP-regulated chloride channel [1]. In addition to its primary biological role, CFTR is likely to affect the expression of a number of gene products, including proteins of the signalling pathways of the inflammatory response [2,3]. One of the major problems of CF is a chronic inflammatory process [4], leading to elevated concentrations of several pro-inflammatory cytokines (i.e. IL-6, TNF-α, IL-1β) and chemokines (i.e. IL-8), released from airway epithelial cells and found in the bronchoalveolar fluid of CF patients [5-8]. The lung inflammation is characterized by a sustained accumulation of neutrophils, high proteolytic activity and elevated levels of cytokines and chemokines, such as interleukin (IL)-8 [9,10]. Massive infiltration of neutrophils in airways, enhanced adherence of neutrophils to CF airway epithelial cells [11,12], elevated levels of cytokines and chemokines in bronchoalveolar lavage fluids such as IL-1β and IL-8 [13,14], may contribute to an over exuberant pro-inflammatory response in lungs of patients with CF.

IL-8 is clearly involved in inflammatory processes associated with CF [15,16]. Interestingly, IL-8 exhibits high release also in CF cells infected with *Pseudomonas aeruginosa *or induced with TNF-α [17].

So far, therapies for cystic fibrosis have been directed at improving airway clearance of secretions and treating endobronchial infection. Recently, there is a growing interest in developing therapies aimed at reducing the excessive inflammatory response in the airways [18,19]. In this respect, extracts from plants used in ethnic medicine are of great interest, as many of them are known to present anti-inflammatory properties [20-29]. For example, Darshan and Doreswamy [26] described the anti-inflammatory activity of drugs derived from 38 medicinal plants. In this review a clear role of botanical plants (including polysaccharides, terpenes, curcuminoids and alkaloids) was reported as alleviating inflammatory diseases, such as arthritis, rheumatic pathologies, acne skin allergy and ulcers.

Bergamot (*Citrus bergamia *Risso) is a typical fruit of the Reggio Calabria province in Southern Italy, where it is mainly used for its essential oil extracted from the peel. Bergamot essential oil is widely used in the pharmaceutical industry because of the antibacterial and antiseptic activity [30] of its volatile fraction, where the major component is the limonene [31]. However, bergamot peel contains also non-volatile components, such as coumarins and furanocoumarins [32]. This is not surprising, since furocoumarins and coumarins are generally distributed throughout the *Citrus *species [33], being the highest amounts are found in *Citrus *peel oils [34]. These compounds are reported to have a broad spectrum of biological activities, including antimicrobial [35], anti-platelet-aggregation [36], anti-mutagenic [37] and anti-inflammatory [38,39] activities.

In the present study, we analyzed the effects of bergamot extracts and their putative isolated compounds on the production of IL-8 in cystic fibrosis IB3-1 and CuFi-1 cells [27,40-42] induced to hyper secretion of pro-inflammatory chemokines following treatment with TNF-α [17,43,44] or exposure to heat-inactivated *Pseudomonas aeruginosa*.

## Materials and methods

### Plant material and extraction procedures

Commercial mature *Citrus bergamia *fruits belonging to three different stocks from organic farming in Southern Italy were purchased and manually processed to completely remove the epicarp. The raw plant material obtained (150 g for each sample stock) were immediately suspended in 600 mL chloroform and processed for the extractions. The suspension was homogenized for 5 min and submitted to sonication in an ultrasound bath (Ultrasonik Mod. 104×, Ney Dental Inc., USA) in the dark at a constant temperature of 25°C. Subsequently, the samples were filtered and centrifuged for 20 min at 3000 *rpm*. The residue was re-extracted with 400 mL chloroform following the same procedure previously described. The collected chloroform extracts, named "extract 1", were partially reduced in volume with Rotavapor and then completely dried under nitrogen flow. The total extraction yield was 3.6 ± 0.2%. Aliquots (5 g) of these sample extracts (extract 1) were subjected to fractional distillation at 350-400 Pa and 40°C, as described elsewhere [45], in order to completely remove volatile terpene compounds, and partly oxygenated chemicals. The sediment obtained at the end of distillation corresponded to the extract 2 samples with an extraction yield of 40.3 ± 0.8%. One g of samples of extract 2 was then re-suspended in 20 mL diethyl ether, vigorously shaken for 30 min and centrifuged. The supernatant was removed and the residue, corresponding to extract 3 samples, was collected, dried under nitrogen flow and weighed (yield: 65.1 ± 1.5%). For all laboratory processing care was taken to protect the operations from light and oxidizing conditions. All the solvents used were purchased from Sigma-Aldrich, Reagent European Pharmacopoeia purity. All the samples (extract 1, extract 2, extract 3) were stored in the dark in glass vials with Teflon-sealed caps at 2.0 ± 0.5°C until analyses by GC-FID, GC-MS, and HPLC to identify and quantify putative bioactive compounds.

### GC-FID and GC-MS analyses

The sample extracts were analyzed and the relative peak areas for volatile and oxygenated components weighted by GC-FID. The relative percentages were determined using GC TRACE Thermoquest, equipped with autosampler Triplus (Thermo Electron Corporation). The column was VF-5ms, 30 m × 0.25 mm. Flow rate was 1.0 mL/min He, and split 1:50. Injector temperature, 300°C; detector temperature, 350°C. Oven temperature was initially 55°C, raised to 100°C at a rate of 1°C/min, and then raised to 250°C at a rate of 5°C/min and finally held at that temperature for 15 min. One μl of each sample (15 mg/mL CH_2_Cl_2_) was injected. The percentage composition of volatile and oxygenated components was computed by the normalization method from the GC peak areas (data integration software: Jasco-Borwin version 1.5, JMBS Developments, Fontaine, France), without correction factors. Identification was performed by a Varian GC-3800 gas chromatograph equipped with a Varian MS-4000 mass spectrometer with electron impact and hooked to a NIST (National Institute of Standards and Technology) library. A Varian FactorFour VF-5ms poly-5% phenyl-95%-dimethyl-siloxane bonded phase column (i.d., 0.25 mm; length, 30 m; film thickness, 0.15 *μ*m) was used. Operating conditions were as follows: injector temperature 300°C; FID temperature 300°C, Carrier (Helium) flow rate 1.0 mL/min and split ratio 1:50. The MS (mass spectrometry) conditions were as follows: ionization voltage, 70 eV; emission current, 10 μAmp; scan rate, 1 scan/s; mass range, 29-400 Da; trap temperature, 150°C, transfer line temperature, 300°C. The main compounds were identified by comparing their relative retention time, KI (Kovats Index) and the MS fragmentation pattern with those of essential oils of known composition, with pure compounds and by matching the MS fragmentation patterns with the above mentioned mass spectra libraries and with those in the literature [18]. To determine the Kovats index of the components, a commercial 24 aliphatic hydrocarbons mixture (Sigma-Aldrich) was added to the sample extracts before injecting in the GC-MS equipment and analyzed under the same conditions as above.

### HPLC analysis

HPLC analysis was performed to quantify the main constituents of the non-volatile fraction of bergamot essential oil. Therefore, pure commercial standards of bergaptol, bergamottin, bergapten, citropten and 5-geranyloxy-7-methoxycoumarin (Extrasynthese, France) were used as external standards to set up and calculate appropriate calibration curves. The experimental conditions were performed using a Jasco modular HPLC (Tokyo, Japan, Model PU 2089) coupled to a Diode Array apparatus (MD 2010 Plus) linked to an injection valve with a 20 μL sampler loop. The column used was a Tracer extrasil ODS2 25 × 0.46 cm, with a flow rate of 1.0 mL/min. The mobile phase employed consisted of the solvent solution B (methanol) and A (water/formic acid = 95/5). The gradient system adopted was characterized by 4 steps: 1. isocratic, with solvent solution B/A = 40/60 (%), for 2 min; 2. the solvent solution B raised progressively from 40 to 60% in 18 min (from min 2 to min 20) until reaching the ratio B/A = 60/40 (%); 3. the solvent solution B then raised 100% in 4 min (from min 20 to min 24); 4. the solvent solution ratio reached B/A = 40/60 in 4 min (from min 24 to min 28). Injection volume was 40.0 μl. Chromatograms were recorded and peaks from bergamot sample extracts were identified by comparing their spectra with spectra obtained with pure standards. Peak area was determined by integration using dedicated Borwin software (Borwin ver. 1.22, JMBS Developments, Grenoble, France). The qualitative and quantitative analyses of each extract were performed three times.

### Cell cultures

IB3-1 cells and CuFi-1 cell lines were obtained from human bronchial epithelium derived from a CF patient with ΔF508del/W1282X mutant genotype (IB3-1 cells) [27] and from a CF patient with a F508del/F508del mutant genotype (CuFi-1 cells) [42]. These cells were grown in LHC-8 basal medium (Biofluids), supplemented with 5% FBS in the absence of gentamycin. All culture flasks and plates were coated with a solution containing 35 mg/ml bovine collagen (Becton-Dickinson), 1 mg/ml bovine serum albumin (Sigma) and 1 mg/ml human fibronectin (Becton-Dickinson). Treatment with TNF-α (80 ng/ml) was performed on 70% confluent cells for 24 hours. Cell number/ml was determined after trypsin treatment by using a model ZBI Coulter Counter (Coulter Electronics, Hialeah, FL, USA). Cells were seeded at the initial concentration of 30,000 cells/cm^2 ^and the cell number/ml determined as IC_50 _after 3 days of culture, when untreated cells are in log phase of cell growth.

### Quantification of IL-8 transcripts

Total RNA was isolated (High Pure RNA isolation kit, Roche), retro transcribed (Promega Corporation, Madison, Wisconsin, USA) and the resulting cDNA was quantified by relative quantitative real-time PCR [46]. The sequences of the oligonucleotides used for amplification of IL-8 mRNA were: 5'-GTG CAG TTT TGC CAA GGA GT-3' (forward) and 5'-TTA TGA ATT CTC AGC CCT CTT CAA AAA CTT CTC-3' (reverse); for β-actin mRNA: 5'-TGA CGG GGT CAC CCA CAC TGT GCC CAT CTA-3' (forward); 5'-CTA GAA GCA TTT GCG GTG GAC GAT GGA GGG-3' (reverse). PCR was performed in a final volume of 50 μl containing 50 mM KCl, 10 mM TRIS-HCl pH 8.8, 1.5 mM MgCl_2 _by using 1U/reaction of Taq DNA polymerase, 100 μM dNTPs, 0.5 μM PCR primers. The 30 PCR cycles used were as follows: denaturation, 30 s, 94°C; annealing, 60 s, 68°C; elongation, 60 s, 72°C. The length of the IL-8 PCR product was 236 bp [17,40]. The marker pUC mix 8 (Fermentas, Milan, Italy) at 0.5 μg/lane was used. For quantitative real-time PCR reaction, 0,5/20 μl aliquots of cDNA were used for each Sybr Green real-time PCR reaction to quantify the relative tissue expression of IL-8 transcripts. Each 25 μl of total reaction volume contained 0.5 μl of cDNA, 10 pmol of primers, 1 × iQ™ SYBR^® ^Green Supermix (Bio-Rad Laboratories, Hercules, CA). Real-time PCR reactions were performed for a total of 40 cycles (95°C for 10 s, 68°C for 30 s, and 72°C for 40 s) using an iCycler IQ^® ^(Bio-Rad Laboratories, Hercules, CA). The relative proportions of each template amplified were determined based on the threshold cycle (Tc) value for each PCR reaction. The ΔΔCt method was used to compare gene expression data. RT-PCR was usually performed in duplicate using two cDNA preparations for each independent experiment. Mean ± S.D. values were determined for each fold difference for at least three independent experiments. Amplification of human β-actin cDNA served as internal standards (housekeeping gene). Duplicate negative controls (no template cDNA) were also run with every experimental plate to assess specificity and i

### IL-8 release

IL-8 in tissue culture supernatants released from the cells under analysis was measured by Bio-Plex cytokine assay (Bio-Rad Laboratories, Hercules, CA) [47,48] as described by the manufacturer. The Bio-Plex cytokine assay is designed for the multiplexed quantitative measurement of multiple cytokines in a single well using as little as 50 μl of sample. In our experiments, the premixed singleplex beads of the Bio-Plex human cytokines IL-8 were used. 50 μl of IL-8 standards or samples (supernatants recovered from treated cells and diluted to 2 μg/μl) were incubated with 50 μl of anti-IL-8 conjugated beads in 96-well filter plates for 30 min at room temperature with shaking. Plates were then washed by vacuum filtration three times with 100 μl of Bio-Plex wash buffer, 25 μl of diluted detection antibody were added, and plates were incubated for 30 min at room temperature with shaking. After three filter washes, 50 μl of streptavidin-phycoerythrin was added, and the plates were incubated for 10 min at room temperature with shaking. Finally, plates were washed by vacuum filtration three times, beads were suspended in Bio-Plex assay buffer, and samples were analyzed on a Bio-Rad 96-well plate reader using the Bio-Plex Suspension Array System and Bio-Plex Manager software (Bio-Rad Laboratories, Hercules, CA).

### Statistical analysis

The statistical significance of difference in between different treatments, was analyzed using one-way analysis of variance (ANOVA) and the Student-Newman Keuls test. *p *values lower than 0.05 were considered significant (*).

## Results

### Effects of bergamot extracts on the expression of IL-8 mRNA in IB3-1 cells following TNF-α treatment: RT-PCR analysis

In order to determine the effects of bergamot extracts on mRNA accumulation, the expression of IL-8 gene was studied by RT-PCR analysis of RNA extracted from TNF-α treated IB3-1 cells cultured in the presence of increasing concentrations of bergamot extracts for 24 hours. These concentrations were chosen according to the IC_50 _values reported in Table 1 and concerning a study of the antiproliferative effects on IB3-1 cells. As target gene, we analysed the accumulation of IL-8 mRNA because IL-8 gene (a) encodes one of the most expressed interleukins in IB3-1 cells [17], (b) it is strongly induced following TNF-α treatment [17], and (c) it is clearly involved in inflammatory processes associated with CF [15,16]. Interestingly, IL-8 exhibits the highest release also in CF cells infected with *Pseudomonas aeruginosa *[40].

**Table 1:** IC_50 _values for bergamot Extracts and relative identified compounds on IB3-1 cell lines.

	**IC**_**50**_
Extract 1	14.5 μg/ml ± 1.5
Extract 2	20.7 μg/ml ± 1.3
Extract 3	30.1 μg/ml ± 3.6
Bergapten	155.8 μM ± 43.6
Bergamottin	43.15 μM ± 6.2
Citropten	235 μM ± 38.2

**Figure 1:**
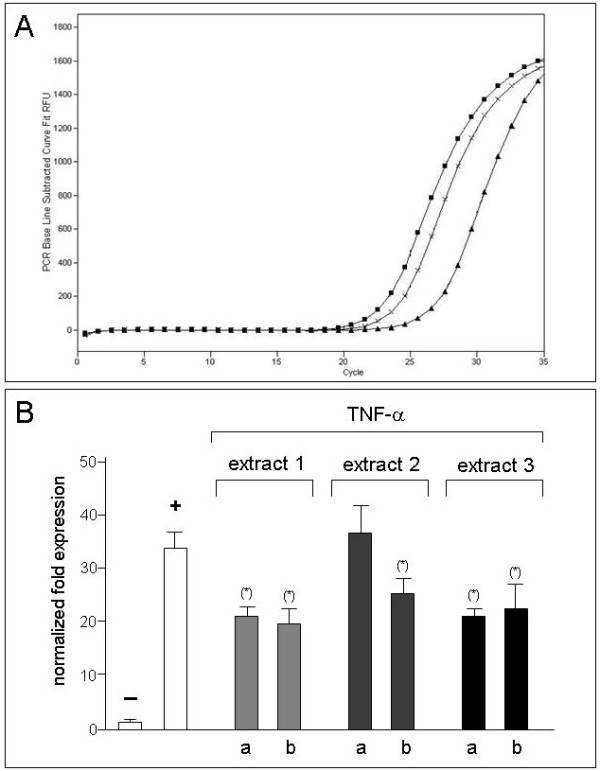
**Effects of bergamot extracts on IL-8 mRNA accumulation**. IB3-1 were treated with ng/ml TNF-α in the presence of increasing concentrations of bergamot extracts for 24 hours, RNA was extracted and RT-PCR performed with primers specific for IL-8 and β-actin RNA sequences. A. Representative quantitative RT-PCR analysis. Triangles: RNA from untreated IB3-1 cells; squares: TNF-α treated IB3-1 cells; crosses: IB3-1 cells treated with IC_50 _extract 3. B: Effects of bergamot extracts on IL-8 mRNA accumulation with respect to β-actin transcripts; (a): extracts added at IC_25_; (b): extracts added at IC_50_. Results represent the average ± S.D. of three independent experiments. For each experiment triplicate RT-PCR determinations were performed. (*) p < 0.05.

Panel A of Figure 1 shows representative results obtained by amplifying RNA from untreated IB3-1 cells (triangles), TNF-α treated IB3-1 cells (squares) or IB3-1 cells treated with TNF-α in the presence of IC_50 _concentration of extract 3 (crosses) using primer specific for IL-8 and β-actin RNA sequences. The β-actin gene was used as reference gene in order to normalized the IL-8 mRNA expression. Clear induction of IL-8 transcripts following TNF-α treatment is evident. Significant inhibitory effects were detected following exposure of TNF-α treated IB3-1 cells to IC_50 _concentration of extract 3.

Panel B of Figure 1 presents the complete set of the results obtained by analysing the real-time quantitative RT-PCR data concerning the effects of bergamot extracts on IL-8 mRNA accumulation with respect to β-actin transcripts. The first set of results consistently demostrate a strong increase of IL-8 mRNA transcript in TNF-α treated IB3-1 cells (average, 34 fold, p < 0.01). In addition, the results obtained indicate that IL-8 mRNA expression is much lower in TNF-α treated IB3-1 cells cultured in the presence of bergamot extracts. More in detail, extract 1 and extract 3, even when used at IC_25 _concentrations, display high level of inhibition of TNF-α induced IL-8 mRNA accumulation. IC_50 _concentrations are necessary to obtain similar inhibitory effects using extract 2.

### Effects of bergamot extracts on the expression of IL-8 genes induced in IB3-1 cells following TNF-α treatment: a Bio-plex analysis

**Figure 2:**
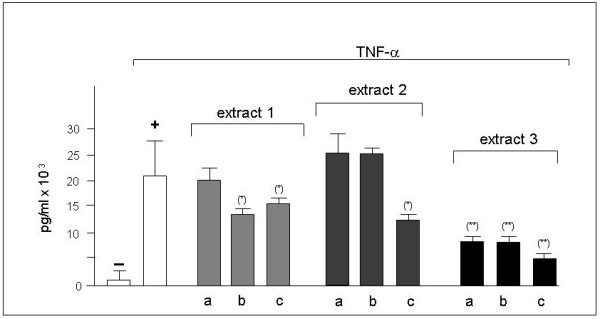
**Effects of bergamot extracts on the IL-8 release**. IB3-1 cells were treated with 80 ng/ml of TNF-α and increasing amounts (a: IC_25_; b:IC_50_; c:IC_75_) of bergamot extracts. After 24 hours, recovered supernatants were analyzed using the Bio-Plex human cytokine IL-8 single-plex (Bio-Rad). (-): untreated IB3-1 cells; (+): IB3-1 cells treated with TNF-α. Results represent the average ± S.D. of three independent experiments. For each experiment duplicate determinations were performed. (*) p < 0.05.

In consideration of the inhibitory activity on IL-8 mRNA accumulation, Bio-plex experiments were performed to verify the possible effects of bergamot extracts on IL-8 release. IB3-1 cells were treated with 80 ng/ml TNF-α in the presence of increasing amounts (IC_25_-IC_50_-IC_75_) of bergamot extracts. After 24 hours, recovered supernatants were analyzed using the Bio-Plex human cytokine IL-8 single-plex (Bio-Rad). When IB3-1 cells were induced with TNF-α and cultured in the presence of bergamot extracts, some important differences in IL-8 release occurred, as reported in Figure 2, i.e. the TNF-α induced release of IL-8 (pg/ml) was strongly reduced using extract 1 and 3 at IC_50 _concentrations. The effect was found to be concentration-dependent. The results obtained indicate a strong inhibition of IL-8 release using extracts 3, even when it was used at IC_25 _concentration, while extract 2 showed inhibitory activity only at IC_75 _concentration. Low level of inhibition was on the contrary detected analyzing the release of VEGF (data not shown). These data suggest that bergamot extracts might specifically inhibit the expression of the IL-8 pro-inflammatory gene.

These data are in agreement with the results obtained using the RT-PCR approach (see Figure 1), and sustain the concept that bergamot extracts are potent inhibitors of the TNF-α induced expression of the IL-8 gene in IB3-1 cells.

### Phytochemical investigation

In consideration of the biological activity shown in Figures 1 and 2, the phytochemical investigation of the three bergamot extracts was performed in order to obtain a qualitative and quantitative determination of the chemical constituents of the extracts, with particular reference to non-volatile fraction chemicals, i.e. coumarins and psoralens. The link between these phytochemical aims and the bioactivity assays targets was to determine the role, if any, of the non-volatile fraction, or of their constituents individually, with or without the synergic interaction of volatile chemicals, in the modulation of IL-8 gene expression in *in vitro *cell models.

**Figure 3:**
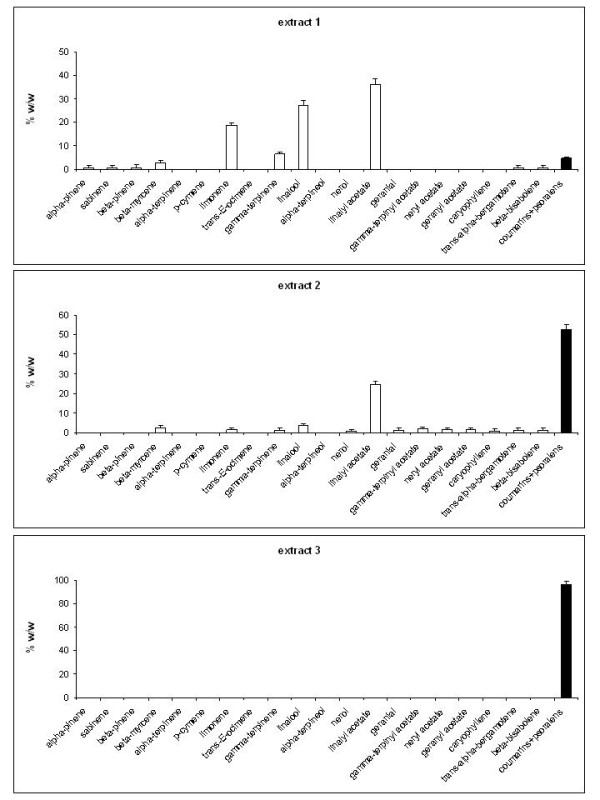
**Phytochemical composition of the bergamot extracts**. The chemical characterizations of bergamot extracts 1 (A), 2 (B) and 3 (C) were performed by GC-MS and GC-FID and expressed as percentage weight/weight [w/w(%)]. The analyses were performed three times.

The chloroform extract (extract 1 samples) composition was similar to that in bergamot essential oil with two significant fractions: a) monoterpenes and their correlated compounds and b) coumarins and psoralens [32]. The extraction procedures followed led to products (extract 2 and extract 3 samples) with a progressive decrease of volatile compounds and an increase of the non-volatile fraction (coumarins and psoralens) as determined by semi-quantitative combined GC-FID and GC-MS analyses (Figure 3). Extract 1 samples showed the presence of linalyl acetate (36.12%), linalool (27.35%) and limonene (18.78%) as the main constituents, as for bergamot essential oil, while coumarins and psoralens were 4.64% (Figure 3). Extract 2 samples showed an important reduction of linalyl acetate (27.45%) and a dramatic decrease of other terpene compounds. Many minority chemicals were not detectable while others, such as limonene, showed a reduction of 93.2%. extract 3 samples gave an even more noticeable reduction of terpene compounds, with linalyl acetate (0.43%), *γ*-terpinene (0.17%) and *β*-myrcene (0.53%) as the only volatile fraction chemicals, against an increase of coumarins and psoralens of 45.5% and 95.2% in the extract 2 and extract 3 samples respectively (Figure 3).

**Figure 4:**
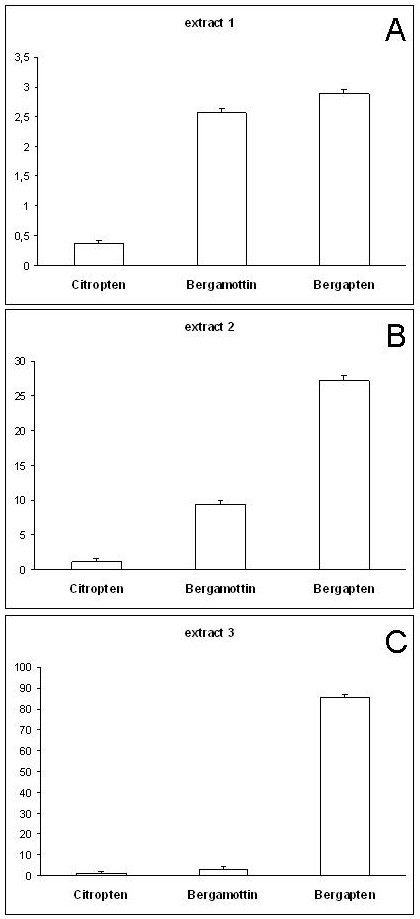
**Characterization of the coumarin/psoralen portion of the bergamot extracts by HPLC analysis**. The percentage (w/w% ± SD) of citropten (5,7-dimethoxycoumarin), bergamottin (5-geranyloxypsoralen) and bergapten (5-methoxypsoralen) were determined for the three bergamot extracts by HPLC analysis.

HPLC analyses were performed to detect and quantify coumarins and psoralens reputed to be representative of the non volatile fraction and of the bioactivity of bergamot crude drug and derived products [32]. Citropten (5,7-dimethoxycoumarin), bergamottin (5-geranyloxypsoralen) and bergapten (5-methoxypsoralen) were identified in all bergamot extract samples. In samples of extract 1 bergamottin and bergapten represented 2.57% and 2.89% respectively, while citropten was detected in lower concentrations (0.37%). In samples of extract 2 concentrations of bergamottin, bergapten and citropten were 9.44%, 27.26%, 1.14%, while in samples of extract 3 concentrations were 3.10%, 85.75% and 0.51% respectively (Figure 4).

In consideration of the fact that extract 3 was the most active on inhibition of TNF-α induced IL-8 mRNA accumulation and IL-8 secretion by treated IB3-1 cells, we hypothesized that the coumarin/psoralen fractions of the extracts were among those responsible for the biological activity. Therefore, the activity of citropten, bergapten and bergamottin have been further analyzed.

### Effects of identified compounds in bergamot extracts on expression of IL-8 genes in TNF-α treated IB3-1 cells

**Figure 5:**
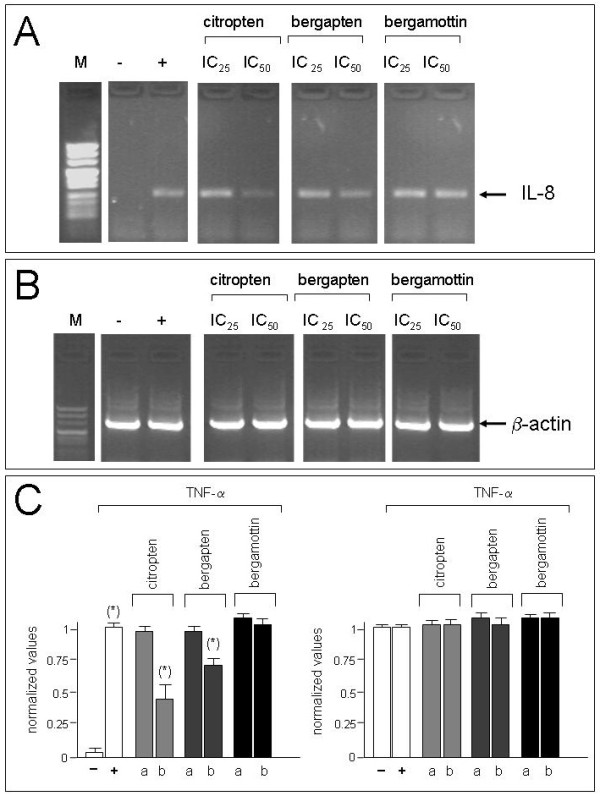
**Effects of citropten, bergamottin and bergapten on IL-8 mRNA accumulation: semi-quantitative RT-PCR analysis**. IB3-1 were treated with TNF-α in the presence of IC_25 _(a) or IC_50 _(b) concentrations of citropten, bergapten and bergamottin as indicated for 24 hours, RNA was extracted and RT-PCR performed with primers specific for IL-8 and β-actin RNA sequences. (-): untreated IB3-1 cells; (+): IB3-1 cells treated with TNF-α. Results represent the average ± S.D. of three independent experiments. For each experiment triplicate RT-PCR determinations were performed. (*) p < 0.05.

In order to determine effects of the three major compounds identified on IL-8 mRNA accumulation, we analyzed, by semi-quantitative RT-PCR, the levels of IL-8 mRNA in TNF-α treated IB3-1 cells [49] cultured for 24 hours in the presence of increasing concentrations of bergamottin, bergapten, citropten (Figure 5). These concentrations were chosen according to IC_50 _values obtained in experiments on anti-proliferative activity determined on IB3-1 cells and reported in Table 1.

The preliminary results obtained indicate that accumulation of IL-8 mRNA is lower in TNF-α treated IB3-1 cells cultured in the presence of bergapten or citropten, administered at IC_50 _concentrations, while bergamottin does not show inhibitory effects at the same concentrations (Figure 5A). The accumulation of β-actin mRNA (used as housekeeping gene) does not change following treatment with bergamot analogues (Figure 5B).

**Figure 6:**
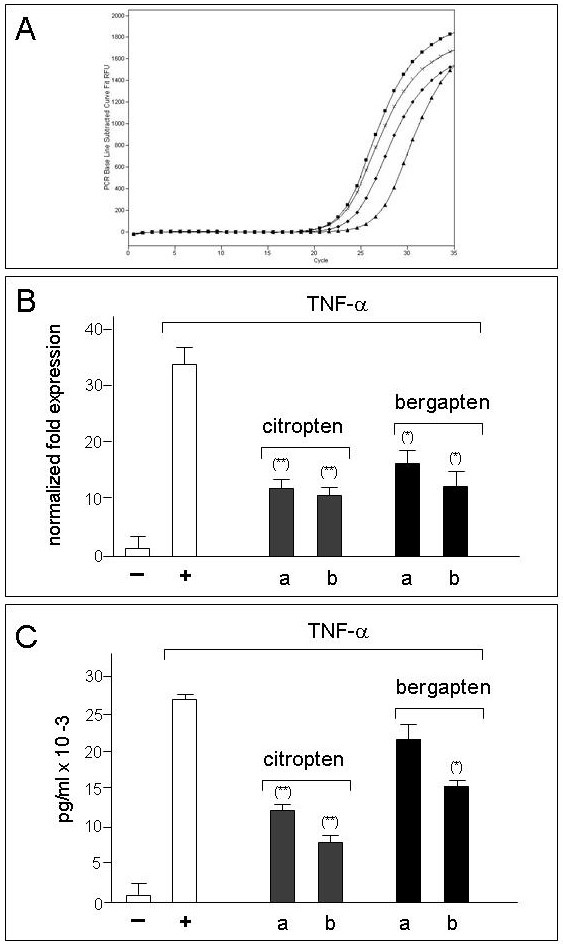
**Effects of citropten and bergapten on TNF-α induced IL-8 gene expression in IB3-1 cells**. A,B: mRNA accumulation studied by quantitative RT-PCR analysis; C: Bio-plex analysis of IL-8 protein present in supernatants. A. representative RT-PCR analysis. triangles: untreated IB3-1 cells; squares: TNF-α treated IB3-1 cells; diamonds: IB3-1 cells treated with citropten; crosses: IB3-1 cells treated with bergapten. B: Summary of the effects of citropten and bergapten on IL-8 mRNA accumulation with respect to β-actin transcripts. B: Bio-plex analysis, using the Bio-Plex human cytokine IL-8 single-plex (Bio-Rad). (-): untreated IB3-1 cells; (+):IB3-1 cells treated with TNF-α. Results represent the average ± S.D. of three independent experiments. For each experiment duplicate RT-PCR determinations were performed. (*) p < 0.05.

On the basis of this first set of results, showing that bergapten and citropten exhibit an interesting inhibitory activity on mRNA accumulation, real-time quantitative RT-PCR analysis was performed to better quantify inhibitory effects of these two compounds on RNA production in IB3-1 cells induced with TNF-α and treated with IC_25 _and IC_50 _concentrations of citropten or bergapten; in these experiments IL-8 mRNA accumulation was compared with that of the internal control β-actin mRNA. Panel A of Figure 6 shows the representative results obtained by amplifying RNA from untreated IB3-1 cells (triangles), or cells treated with TNF-α (squares), in the presence of citropten (diamonds, IC_50 _concentration) and bergapten (crosses, IC_50 _concentration), using primers specific for IL-8 RNA. Clear induction of IL-8 transcripts following TNF-α treatment is evident, but this increase is lower in the presence of treatment with citropten or bergapten. Citropten was found to be more efficient than bergapten in inhibiting IL-8 mRNA accumulation. This result is more evident in Panel B of Figure 6, showing a summary of the data obtained by real-time quantitative RT-PCR performed to investigate the effects on IL-8 mRNA accumulation of bergapten and citropten at two different concentrations (IC_25 _and IC_50_). Significant inhibitory effects on IL-8 mRNA accumulation were detected following exposure of TNF-α induced IB3-1 cells to citropten and bergapten, even when they were administered at IC_25 _concentrations. Moreover, citropten was found to be more active than bergapten at the same concentrations. These data are in agreement with data obtained by semi-quantitative RT-PCR (see Figure 5), and confirm the potent inhibitory activity of citropten and bergapten on expression of the IL-8 gene in IB3-1 cells.

The results shown in Figure 6B have been also confirmed using Bio-plex technology to analyze the release of IL-8 protein (pg/ml) in supernatants recovered from IB3-1 cells induced with TNF-α and treated with citropten and bergapten at IC_50 _and IC_25 _concentrations. These supernatants were collected before performing the RNA extraction for real time PCR analysis. The results, shown in Figure 6C, confirmed the inhibitory activity of citropten and bergapten (again citropten was found to be more active than bergapten) on expression of IL-8 protein in IB3-1 cell model. No inhibitory effects on VEGF release were found (data not shown).

### Effects of bergapten and citropten on IB3-1 cells based experimental system mimicking Pseudomonas aeruginosa infection

**Figure 7:**
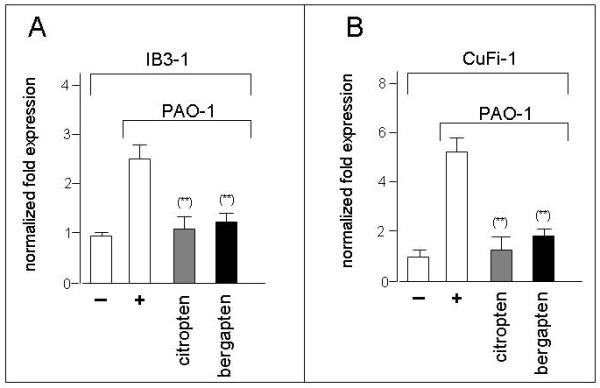
**Effects of citropten and bergapten on IB3-1 and CuFi-1 cells**. mRNA accumulation was studied by quantitative RT-PCR analysis. IL-8 expression was induced by exposure of IB3-1 (A) and CuFi-1 (B) to heat-inactivated PAO-1. White symbols: untreated IB3-1 or CuFi-1 cells, in the absence (-) or in the presence (+) of heat-inactivated PAO-1. Grey symbols, cells treated with IC_25 _bergapten; Black symbols, cells treated with IC_25 _citropten. Results represent the average ± S.D. of three independent experiments. For each experiment duplicate RT-PCR determinations were performed. (*) p < 0.05.

In order to determine the effects of bergapten and citropten on an experimental system resembling more closely the cystic fibrosis disease, we employed IB3-1 exposed to heat-inactivated *Pseudomonas aeruginosa*, strain PAO-1. This induces an increase of cytokines and chemokines, mimicking the infection of *P.aeruginosa*, which is an important step in generating the clinical symptoms of CF, as reported in several studies and reviews [50,51] Figure 7A show that exposure of IB3-1 to heat-inactivated PAO1 causes an increase of IL-8 mRNA production, as elsewhere reported. In agreement with the data reported in Figure 5, treatment of IB3-1 cells exposed to PAO-1 leads to sharp inhibition of IL-8 gene expression.

### Effects of bergapten and citropten on CuFi-1 cystic fibrosis cells

In Figure 7B, the experiments conducted with a second CF cell line, CuFi-1, are reported. CuFi-1 cells were exposed to PAO-1, in the absence or in the presence of bergapten or citropten. Induction of IL-8 mRNA accumulation was obtained after exposure of CuFi-1 cells to PAO-1 (Figure 7B) and IL-8 gene expression was inhibited by bergapten and citropten.

## Discussion

One of the clinical feature of cystic fibrosis (CF) is a deep inflammatory process, which is characterized by production and release of cytokines and chemokines, among which interleukin 8 (IL-8) represent one of the most important [15,16]. In fact, high levels of IL-8 are present in CF cells infected with *Pseudomonas aeruginosa *or induced with TNF-α [17]. Accordingly, there is a growing interest in developing therapies for CF in order to reduce the excessive inflammatory response in the airways [18,19] of CF patients. In this respect, extracts from medicinal plants have been reported to exhibit anti-inflammatory activities in several reports [20-29].

The major result of this article is the finding that extracts obtained from bergamot (*Citrus bergamia *Risso) epicarps, contain components displaying an inhibitory activity on IL-8 gene expression. This effect has been confirmed both at the mRNA levels and at the protein release level in the CF cellular model IB3-1, induced with TNF-α and treated with low concentrations of bergamot extracts.

The qualitative and quantitative determination of the chemical constituents of the extracts, with particular reference to non-volatile fraction chemicals, has demonstrated the presence of citropten, bergamottin and bergapten in all bergamot extract samples despite with different amounts (%w/w) (see Figures 3 and 4). In detail, the coumarin/psoralen portion was the major constituent of the most active extract (extracts 3). Accordingly, we hypothesized that the coumarin/psoralen portion of these extracts was responsible of the biological activity.

On the other hand, the data reported in the present paper do not clarify why extract 2 exhibited the lower biological activity. It should be noted that the composition of extract 1 and extracts 2 is complex and, therefore, the presence of compounds exhibiting opposite activity or interfering with the coumarin/psoralen compounds cannot be excluded.

A second achievement of the results here presented is the demonstration that, among the prominent coumarin/psoralen compounds, bergapten and citropten were the most active molecules in reducing IL-8 mRNA levels in TNF-α treated IB3-1 cells at IC_50 _concentrations, while bergamottin did not show any inhibitory effects at the same (see Figure 5) and higher doses (data not shown). These results have been confirmed by real-time quantitative RT-PCR and Bio-plex analyses. Citropten is more efficient than bergapten in decreasing IL-8 mRNA/protein accumulation in IB3-1 cells.

The effects of bergapten and citropten on IL-8 mRNA accumulation were confirmed in IB3-1 exposed to heat-inactivated PAO-1, mimicking *Pseudomonas aeruginosa *infection [50]. In addition the effects of bergapten and citropten on IL-8 mRNA accumulation have been confirmed using another CF cell line, CuFi-1.

## Conclusion

The results obtained in this study clearly indicate that bergapten and citropten are strong inhibitors of IL-8 expression and could be proposed as potential anti-inflammatory molecules to reduce lung inflammation in CF patients. Further experiments are required to: (a) identify other biochemical targets of citropten and bergapten, as well as whether the inhibitory effects on IL-8 gene expression are reproducible in other cellular pathological systems in which this chemokine plays a role; (b) determine safety, potential toxicity, *in vivo *bioavailability, stability and whether suitable concentrations can be reached following in vivo administration and (c) *in vivo *anti-inflammatory effects. We would like to point out that our *in vitro *study does not guarantee for anti-inflammatory activity *in vivo*. However, published results support the concept that molecules affecting the expression of IL-8 *in vitro *on the IB3-1 system display also anti-inflammatory activity *in vivo*. For instance, Bergamini et al. [52] found that exposure of IB3-1 cells to Azitromycin (AZT) significantly decreased gamma-glutamyltransferase (GGT) activity, restoring the levels to those observed in non-CF cells. Interestingly, in bronchoalveolar lavage fluid of CF mice homozygous for the F508 del mutation, GST expression was undetectable, suggesting novel antioxidant properties for this drug. Another example are decoy oligonucleotides against NF-kB, which are potent inhibitors of IL-8 gene expression in *Pseudomonas aeruginosa *infected IB3-1 cells [53]. These decoys against NF-kB were found to reduce chronic inflammation in rats [54].

Moreover, it should be considered that coumarins compounds, as reported in literature, display anti-proteinase activity [55]. For example, serin proteinase is capable to degradate the matrix proteins and is involved in several inflammatory respiratory diseases as CF. It will be very interesting to investigate and correlate the anti-inflammatory effect of bergapten and citropten with their possible anti-proteinase activity. This double actions could be useful for the treatment of lung inflammation in CF patients.

## Abbreviations

(CF): Cystic fibrosis; (NMR): nuclear magnetic resonance; (GC-FID): gas chromatography-flame ionization detector; (GC-MS): gas chromatography-mass spectrometry; (HPLC): high pressure liquid chromatography; (TNF): tumor necrosis factor; (CFTR): cystic fibrosis transmembrane conductance regulator; (FBS): fetal bovine serum; (PCR): polymerase chain reaction; (IL): interleukin.

## Competing interests

The authors declare that they have no competing interests.

## Authors' contributions

MB and IM carried out the real-time PCR and Bio-plex analyses; NB and IL performed cell culture and semi-quantitative PCR; AG and DR carried out extraction and chemical characterization of extracts; GS and RG supervised the experimental work and drafted the manuscript. All authors read and approved the final manuscript.

## Acnowledgements

Roberto Gambari has received grants from AIRC, Fondazione CARIPARO (Cassa di Risparmio di Padova e Rovigo), Cofin-2002, Telethon (contract GGP10214). Monica Borgatti has received a 2006 Young Investigator Grant from the Ferrara University, Italy. This work was also supported by contributions from FFC (Italian Cystic Fibrosis Research Foundation) to R.G. (FFC#17/2010) and to M.B. (FFC#2/2010). This research is also supported by The Emilia-Romagna Region and by Associazione Veneta per la Lotta alla Talassemia, Rovigo.
